# Dementia Knowledge and Caregiving Skills Improvement From Using the PAC Model: A Pilot Comparative Evaluation

**DOI:** 10.1093/geroni/igaa057.293

**Published:** 2020-12-16

**Authors:** Jerry Brown, Pranay Reddy, Candidus Nwakasi, Beth Nolan, Teepa Snow, Katie Ehlman

**Affiliations:** 1 University of Southern Indiana, Evansville, Indiana, United States; 2 Positive Approach to Care, Ada, Michigan, United States; 3 Teepa Snow, Efland, North Carolina, United States

## Abstract

The diverse needs of persons living with dementia in nursing home settings presents challenges for Certified Nursing Assistants (CNAs) to provide quality care. There is a lack of educational preparedness among nursing home CNAs regarding dementia knowledge and skills required to care for a person living with dementia. As direct caregivers for persons living with dementia, CNAs play an important role in long-term care. This pilot study evaluated the dementia knowledge and caregiving skills of newly trained CNA students. The students were trained by an instructor certified using Teepa Snow’s Positive Approach to Care (PAC) curriculum. Conducted in a rural southwestern Indiana community, this study evaluated CNA students’ knowledge and perception of dementia, as well as their skill performing the Positive Physical Approach™ (PPA™) technique to approach and connect. A 38-item knowledge and perception survey and a 12-step observed skills assessment using a standardized patient encounter were administered to CNA students. Data were analyzed using descriptive statistics and bivariate analysis. Preliminary results indicate that 100% of students correctly answered the survey item regarding non-confrontational body language, while 29% of students correctly performed the corresponding PPA skill. There is a statistically significant association between the knowledge that people find pressure in their palm comforting and the ability to perform the corresponding Hand-under-Hand® and PPA techniques. Incorporation of PAC into current CNA curriculum may equip CNAs with the knowledge and skills required to provide better care, with the potential to improve the overall quality of life for persons living with dementia.

